# Paternal leakage and mtDNA heteroplasmy in *Rhipicephalus* spp. ticks

**DOI:** 10.1038/s41598-018-38001-8

**Published:** 2019-02-06

**Authors:** Valentina Mastrantonio, Maria Stefania Latrofa, Daniele Porretta, Riccardo Paolo Lia, Antonio Parisi, Roberta Iatta, Filipe Dantas-Torres, Domenico Otranto, Sandra Urbanelli

**Affiliations:** 1 Department of Environmental Biology, Sapienza University of Rome, Rome, Italy; 2 Department of Veterinary Medicine, University of Bari, 70010 Valenzano, Bari Italy; 3 Istituto Zooprofilattico Sperimentale della Puglia e della Basilicata, Contrada S. Pietro Piturno, 70017 Putignano, Bari, Italy; 4 Department of Immunology, Aggeu Magalhães Institute, Oswaldo Cruz Foundation, 50740465 Recife, Pernambuco Brazil

## Abstract

Paternal leakage of mitochondrial DNA (mtDNA) and heteroplasmy have been recently described in several animal species. In arthropods, by searching in the Scopus database, we found only 23 documented cases of paternal leakage. Therefore, although arthropods represent a large fraction of animal biodiversity, this phenomenon has been investigated only in a paucity of species in this phylum, thus preventing a reliable estimate of its frequency. Here, we investigated the occurrence of paternal leakage and mtDNA heteroplasmy in ticks belonging to one of the most significant tick species complexes, the so-called *Rhipicephalus sanguineus* *sensu lato*. By developing a multiplex allele-specific PCR assay targeting a fragment of the 12S rRNA ribosomal region of the mtDNA, we showed the occurrence of paternal leakage and mtDNA heteroplasmy in *R. sanguineus* *s.l.* ticks originated from experimental crosses, as well as in individuals collected from the field. Our results add a new evidence of paternal leakage in arthropods and document for the first time this phenomenon in ticks. Furthermore, they suggest the importance of using allele-specific assays when searching for paternal leakage and/or heteroplasmy, as standard sequencing methods may fail to detect the rare mtDNA molecules.

## Introduction

Mitochondrial DNA (mtDNA) has long been considered an optimal marker in population genetics, phylogenetics and phylogeographic studies for both vertebrates and invertebrates^1,2^. A fragment of the gene encoding for the cytochrome oxidase subunit 1 (*cox*1) has been even used for the barcoding of animal species^3–5^. The success of mtDNA as a molecular marker has been associated to some features generally attributed to it, such as high mutation rate, maternal inheritance and absence of recombination^1^.

Maternal inheritance of mtDNA has been deemed as a rule in animal species and several stochastic and deterministic molecular mechanisms preventing the transmission of paternal mtDNA to the zygote have been described^6–10^. However, the transmission of the male parent’s mitochondria to the offspring (i.e., paternal leakage) has been observed in an increasing number of taxa^10,11^. An outcome of paternal leakage is a state of heteroplasmy in the progeny, where both paternal and maternal mtDNA are present within an individual^10^. Cases of heteroplasmy due to paternal leakage have been revealed in several animals, such as fishes, reptiles, birds and mammals^10,11^, which is calling into question the assumption of strict maternal inheritance. Notably, mtDNA heteroplasmy could be higher than currently recognized, since the commonly used techniques, such as standard PCR-amplification and Sanger sequencing, are not always able to reveal mtDNA molecules occurring at low frequencies^10,12^. Furthemore, mtDNA leakage might be more frequent than currently observed because it is only detectable when the parental mtDNA genomes are so different to be recognized. As consequence, although it appears to be more common in interspecific crosses, paternal leakage may be common after crosses between conspecific individuals belonging to genetically divergent lineages^10,11,13^. Undoubtedly, additional studies about the frequency of paternal leakage among different species would be valuable, as this phenomenon could eventually affect the inferences about the evolutionary history of species or populations based on mtDNA data^14^.

Although arthropods represent a large fraction of animal biodiversity and include several species of ecological, socio-economical and medical-veterinary importance^15–19^, to date the occurrence of paternal leakage and mtDNA heteroplasmy has been documented only in a paucity of species . Fontaine *et al*.^13^ reviewed the cases of paternal leakage in animals up to 2007 and revealed that only 11 cases were documented in arthropod species. By searching for “paternal leakage AND animals” in the Scopus database since 2007, we found 31 papers and, among them, 10 new cases in arthropod species (up to 30^th^ October 2018) (Table 1). Given the large number of arthropods that remain to be screened, the frequency of paternal leakage may be currently underestimated.

**Table 1:** Cases of paternal leakage in arthropod species.

Common name		Reference
**Heterospecific crosses**		
Fruit flies	*Drosophila yakuba* × *Drosophila mauritiana*	^47^
Fruit flies	*Drosophila teissieri* × *Drosophila mauritiana*	^47^
Fruit flies	*Drosophila simulans* × *Drosophila mauritiana*	^47^
Fruit flies	*Drosophila simulans* × *Drosophila sechellia*	^47^
Periodical Cicada	*Magicicada septendecim* × *M. cassini*	^13^
Periodical Cicada	*Magicicada septendecim* × *M. septendecula*	^13^
Silkmoth	*Antheraea pernyi* × *A. roylei*	^48^
Fruit flies	*Drosophila mauritiana* × *D. simulans*	^49^
Fruit flies	*Drosophila mauritiana* × *D. simulans*	^50^
Tobacco budworm	*Heliothis virescens* × *H. subflexa*	^51^
**Conspecific crosses**		
Onionthrips	*Thrips tabaci*	^52^
Leafbeetle	*Gonioctena intermedia*	^53^
Bed bug	*Cimex lectularius* L.	^45^
Fruit fly	*Drosophila simulans*	^22^
Fruit fly	*Drosophila melanogaster*	^44^
Fruit fly	*Drosophila mauritiana*	^49^
Fruit fly	*Drosophila simulans*	^49^
Scorpion	*Buthus mardoechi*	^54^
Scorpion	*Mesobuthus caucasius*	^54^
Scorpion	*Mesobuthus eupeus*	^54^
Scorpion	*Mesobuthus gibbosus*	^54^
Eastern tiger swallowtail	*Papilio glaucus*	^55^
Honeybee	*Apis mellifera*	^56^

In this paper, we aimed to contribute to fill this gap of knowledge. In ticks, the occurrence of paternal leakage has been recently hypothesized in the brown dog ticks (*Rhipicephalus sanguineus* *sensu lato*), as crossbreeding experiments between two temperate lineages of *R. sanguineus* *s.l.*, namely *Rhipicephalus* sp. I (*R*. sp. I) and *Rhipicephalus* sp. II (=*Rhipicephalus sanguineus* *sensu stricto*), showed offspring individuals harbouring paternal mtDNA^20^. Here, we assessed whether paternal leakage and heteroplasmy occur in *R. sanguineus* *s.l.*, by analysing *R. sanguineus* s.s and *R*. sp. I individuals originated from experimental crosses^20^, as well as wild-caught ticks coming from the same areas where parental individuals used in the aforementioned crossbreeding experiments were collected. The occurrence of heteroplasmy in *R. sanguineus* *s.l.* ticks was screened by a newly developed multiplex allele-specific PCR assay (MAS-PCR) targeting a fragment of the 12S rRNA ribosomal region in the mtDNA.

## Results

### Multiplex allele-specific polymerase chain reaction reliability

**Figure 1:**
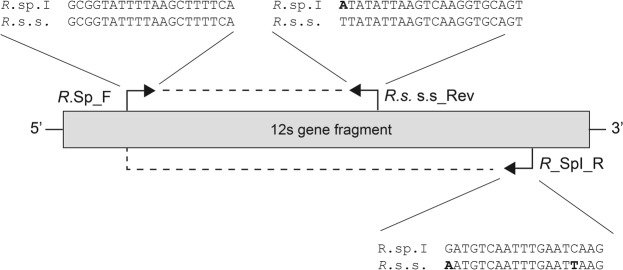
Schematic representation of the primer pairs used to amplify the 12S rRNA gene fragment. Primer pair *R*.sp._For/*R*.sp. I_Rev: PCR product of 270 bp; Primer pair *R*.sp._For/*R. s.* s.s._Rev: PCR product of 160 bp.

**Figure 2:**
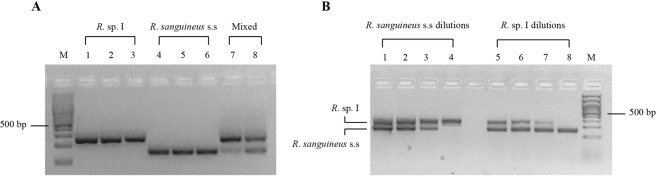
Specificity and sensitivity tests of MAS-PCR assay. (**A**) Lines 1–3: Electrophoretic pattern of known samples of *R*. sp. I, Lines 4–6: *R. sanguineus* s.s., Lines 7–8: mixed DNA (*R*. sp. I + *R. sanguineus* s.s.), M: 100 bp DNA ladder. (**B**) Electrophoretic pattern of mixed DNA between *R*. sp. I and *R. sanguineus s.s*. diluted at different ratios. Starting DNA concentration 5 ng/µl. Lines 1–4: ratios 1:10, 1:50, 1:100, and 1:1.000. Lines 5–8: ratios (1:10), (1:50), (1:100), (1:1.000). M: 100 bp DNA ladder. The gels in (**A**) and (**B**) are different gels. The original photos of the gels are shown in the Supplementary information.

The MAS-PCR was designed to amplify a mitochondrial fragment of the 12S rRNA of *R*. sp. I and *R. sanguineus* s.s. (Fig. 1) for which a genetic divergence of about 10% was recorded^21^. The specificity of the MAS-PCR was assessed using known *R*. sp. I and *R. sanguineus* s.s. individuals and mixed genomic DNA. Electrophoretic bands of 270 bp and 160 bp were observed for *R*. sp. I and *R. sanguineus* s.s., respectively, as well as two bands for the mixed DNA sample (Fig. 2A). The specificity of the amplification was confirmed by the sequences of the amplicons and the alignment with the reference sequences of *R*. sp. I and *R. sanguineus* s.s. available in Genbank (KC243791.1-KC243807.1). The sensitivity of the MAS-PCR, assessed by amplifying serially diluted mixed DNA of both lineages at known concentrations, showed a detectable amount as low as 0.05 ng (Fig. 2B).

### Screening of tick individuals

Crosses were performed by using *R*. sp. I and *R. sanguineus* s.s. ticks from Italy and Portugal^20^. A total of 80 parental individuals and 160 F1 offspring were then screened for paternal leakage and heteroplasmy by MAS-PCR. According to this assay, homoplasmic individuals were expected to show a single electrophoretic band of 270 and 160 base pairs for *R*. sp. I and *R. sanguineus* s.s., respectively. On the contrary, both bands are expected for heteroplasmic individuals after the electrophoretic run.

**Figure 3:**
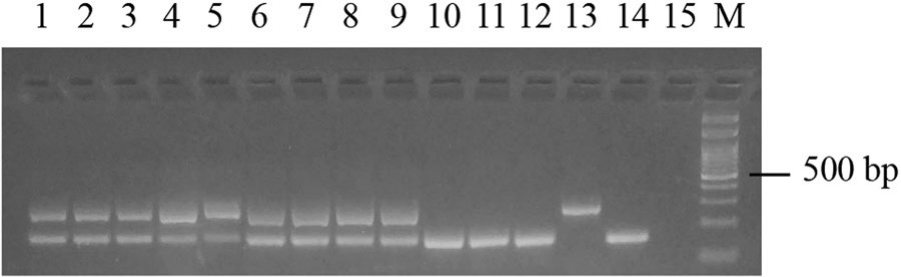
*Rhipicephalus sanguineus* *s.l.* individuals showing paternal mtDNA using MAS-PCR. Lines 1–3: heteroplasmic parental *R*. sp. I female, F1 larva and F1 nymph from the experimental cross ♀ *R*. sp. I × ♂ *R*. sp. I. Lines 4–6: heteroplasmic F1 larvae and F1 adult female from the experimental cross ♀ *R*. sp. I × ♂ *R. sanguineus* s.s. Lines 7–9: heteroplasmic females from Putignano. Lines 10–12: F1 larvae from the experimental cross ♀ *R*. sp. I × ♂ *R. sanguineus* s.s. showing paternal mtDNA. Lines 13–14: positive control for *R*. sp. I and *R. sanguineus* s.s., respectively. Line 15: negative control. M: DNA ladder 100 bp. The original photo of the gel is shown in the Supplementary information.

All parental individuals used to originate the crosses showed the expected electrophoretic banding pattern, with the exception of one *R*. sp. I female, which was found to be heteroplasmic (Table 2). In the offspring originating from the pure crosses, all F1 individuals from the cross “♀ *R. sanguineus* s.s. × ♂ *R. sanguineus* s.s.” showed the maternal mtDNA, as expected. On the contrary, among the progenies originated from the cross “♀ *R*. sp. I × ♂ *R*. sp. I”, two F1 heteroplasmic individuals were found, that showed both bands in the electrophoretic pattern (i.e., 160 and 270 bp bands) (Fig. 3). In the cross “♀ *R. sanguineus* s.s. × ♂ *R*. sp. I”, all F1 individuals showed the maternal mtDNA, while in the cross “♀ *R*. sp. I × *R. sanguineus* s.s.”, three F1 individuals showed the 160 bp band (i.e., paternal mtDNA) and three individuals showed both the 160 and 270 bp bands (i.e., maternal and paternal mtDNA) (Table 2, Fig. 3).

**Table 2:** 12S mitochondrial DNA genotypes of the *Rhipicephalus* sp. I and *R. sanguineus* s.s. individuals analysed by multiplex allele-specific PCR approach.

	12S mtDNA genotype		
	*R*. sp. I	*R. sanguineus s.s*.	Heteroplasmic
**Experimental crosses**			
♀ *R*. sp. I × ♂ *R*. sp. I			
Parental *R*. sp. I females	9	—	1
Parental *R*. sp. I males	10	—	—
F1, larvae	4	—	1
F1, nymphs	4	—	1
F1, adult females	5	—	—
F1, adult males	5	—	—
♀ *R. sanguineus* s.s. × ♂ *R. sanguineus* s.s.			
Parental *R. sanguineus* s.s. females	—	10	—
Parental *R. sanguineus* s.s. males	—	10	—
F1, larvae	—	5	—
F1, nymphs	—	5	—
F1, adult females	—	5	—
F1, adult males	—	5	—
♀ *R. sanguineus* s.s. × ♂ *R*. sp. I			
Parental *R. sanguineus* s.s. females	—	10	—
Parental *R*. sp. I males	10	—	—
F1, larvae	—	10	—
F1, nymphs	—	10	—
F1, adult females	—	10	—
F1, adult males	—	10	—
♀ *R*. sp. I × ♂ *R. sanguineus* s.s.			
Parental *R*. sp. I females	10	—	—
Parental *R. sanguineus* s.s. males	—	10	—
F1, larvae	5	3	2
F1, nymphs	10	—	—
F1, adult females	9	—	1
F1, adult males	10	—	—
**Natural populations**			
Putignano (Italy)			
Larvae	5	—	—
Nymphs	5	—	—
Females	2	—	3
Males	5	—	—
Faro (Portugal)			
Larvae	—	5	—
Nymphs	—	5	—
Females	—	5	—
Males	—	5	—

By analysing wild-caught ticks, all individuals collected from Portugal presented the160 bp band. On the contrary, three out of the 20 *R*. sp. I individuals analysed from southern Italy showed bands of both sizes (160 bp and 270 bp) (Table 2, Fig. 3).

In all heteroplasmic individuals the two parental alleles were found in a similar ratio (Fig. 3). No bands were observed in any negative control included in PCR reactions using laboratory and field samples.

Sequencing of the PCR products from a subset of parental individuals (five females and five males from each cross), from all individuals with the paternal mtDNA type and from all heteroplasmic individuals, confirmed the genetic identity of ticks (i.e., 270 bp PCR product identical to the *R*. sp. I haplotype_h4 KC243794.1, and the 160 bp PCR product identical to *R. sanguineus* s.s. haplotype_h1KC243802.1).

## Discussion

The occurrence of paternal leakage and mtDNA heteroplasmy was hypothesized in a previous study involving *R*. sp. I and *R. sanguineus s.s*. ticks, as individuals carrying paternal mtDNA were observed after experimental crosses^20^. The results here obtained supported the above hypothesis, by showing the occurrence of paternal leakage and mtDNA heteroplasmy in *R. sanguineus* *s.l.* ticks. The MAS-PCR assay showed the occurrence of heteroplasmic ticks and individuals carrying paternal mtDNA among the offspring of the crosses, as well as in individuals collected from southern Italy (Table 2). Misleading results due to unspecific PCR amplification of the mtDNA of the two species or to the amplification of non-functional nuclear copies of mitochondrial genes (NUMTs) can be ruled out. The MAS-PCR assay, indeed, has proved to be highly specific (Fig. 2). Furthermore, consistent results between MAS-PCR and the sequencing of PCR products were observed.

In the cross “♀ *R*. sp. I × ♂ *R*. sp. I”, a previous study using standard PCR-amplification and sanger sequencing^20^, showed that two F1 individuals carried the *R. sanguineus* s.s. mtDNA, although all parental individuals carried the *R*. sp. I mtDNA^20^. Here, the use of the MAS-PCR assay allowed us to explain this unexpected result, by showing the presence of a single heteroplasmic female among the parental ticks used to originate this cross, that standard techniques were not able to detect (Table 2).

Paternal leakage was observed in the cross “♀ *R*. sp. I × ♂ *R. sanguineus* s.s.”, but not in the reciprocal cross. In the cross “♀ *R. sanguineus* s.s. × ♂ *R*. sp. I” paternal leakage could be intrinsically prevented. In animals, indeed, paternal leakage of mtDNA is usually prevented by several stochastic and molecular mechanisms that efficiently exclude paternal mtDNA (e.g., dilution of paternal mtDNA by female mtDNA in the zygote, selective tagging of paternal mtDNA, destruction upon fertilization, bottleneck in the early stages of embryogenesis)^7,10,11^. Alternatively, it simply did not occur in this cross by chance or it could not be detected by our MAS-PCR assay. Interestingly, in the cross “♀ *R*. sp. I × ♂ *R. sanguineus* s.s.”, three out of the F1 individuals analysed carried only paternal mtDNA, with no evidence of maternal mtDNA (Table 2, Fig. 3). In these individuals, maternal mtDNA levels could be below the detection capacity of our assay or, alternatively, complete paternal replacement may have occurred^10,11,14^. This phenomenon has been recently reported in the fruit fly *Drosophila simulans*^22^. In this species, different factors have been hypothesized to explain paternal replacement, such as positive selection in favour of the paternal haplotype, replicative advantage or environmental factors that may impact the inheritance and propagation of paternal mtDNA (i.e., temperature), and stochastic processes due to bottleneck effect or random assortment of parental mtDNA during the early developmental stages^22^. In *R. sanguineus* *s.l.* ticks, a role of the environmental factors, such as temperature can be excluded, as it was kept constant in our experiments. Considering that the inheritance of paternal mtDNA could impact not only on the evolution of the molecule but also affect species or population fitness^23,24^, specific studies addressing the individual fitness will allow us to assess the relative roles of selective and stochastic processes in the observed pattern.

The results from the crossbreeding experiments corroborated those obtained with wild-caught ticks, indeed, mtDNA heteroplasmy was found in *R*. sp. I individuals from Putignano. This result could suggest the possible occurrence of hybridization between *R*. sp. I and *R. sanguineus* s.s. in nature with subsequent paternal leakage-driven mtDNA heteroplasmy. Hybrid zones, where divergent genetic lineages meet and exchange genes^25–31^, have been often associated with paternal leakage and mtDNA heteroplasmy, as the molecular mechanisms preventing paternal mtDNA inheritance may not be efficient between hybridizing species or populations, due to the high genetic divergence between parental lineages^10,11^. Interestingly, sympatric areas have been reported between *R*. sp. I and *R. sanguineus* s.s. ticks, since both lineages were found in Serbia, Algeria, and in southern and central Italy^21,32–34^. In this paper, we analysed one population from southern Italy, that allowed us to detect the occurrence of mtDNA heteroplasmy in *Rhipicephalus* spp. ticks. A more intense sampling of the Italian populations, as well as of the other sympatric areas will allow us to estimate how frequent this phenomenon is in nature.

The occurrence of paternal leakage and mtDNA heteroplasmy may affect taxonomy and systematic inferences based on mtDNA data alone, because it may lead to misleading identification of individuals. This may be particularly relevant in areas where different species coexist, such as secondary contact zones, or areas of recent colonization. Furthermore, heteroplasmy can lead to recombination between heterologous mtDNA molecules, which, if unaccounted for, can affect the interpretation of phylogenetic reconstruction as well as the population demographic histories inferred by mtDNA data^10,11,14,35,36^. As far as *R. sanguineus* *s.l.* ticks, the finding of paternal leakage suggests that multiple approaches are recommended, such as the use of both nuclear and mitochondrial genetic markers and of specific assays to detect the potential occurrence of heteroplasmy.

MtDNA heteroplasmy due to mutation in the mitochondrial genome sequence has been described in different arthropod species, including ticks^37–43^. On the contrary, mtDNA heteroplasmy due to paternal leakage has been documented only in a paucity of arthropod taxa, and at our best knowledge, it has never been described in ticks to date. Our finding, therefore, represents the first evidence of this phenomenon in ticks and adds to the few examples of paternal leakage and mtDNA heteroplasmy described in arthropods^13,22,44,45^. Notably, as the commonly used PCR-Sanger sequencing approach can fail to detect mitochondrial heteroplasmy^44,45^, studies aimed to search for paternal leakage and mtDNA heteroplasmy in hybrid zones using specific assays are desirable to really appreciate how frequent they are in nature, as well as to assess their evolutionary relevance.

## Materials and Methods

### MAS-PCR

A MAS-PCR assay was designed on the 12S rRNA region of mtDNA^21^. Reference sequences of the 12S rRNA gene fragment (347 bp) of *R*. sp. I and *R. sanguineus* s.s. were used to design the primers for our MAS-PCR assay^21^. The software Primer3Plus, that identifies a list of possible primer pairs, and Beacon designer, that allows checking for cross homologies and template structures during the primer design, were used. We designed: *i*) a *common forward primer* for *R*. sp. I and *R. sanguineus* s.s. in a conserved region of the 12S gene (*R*.sp._For, 5′-GCGGTATTTTAAGCTTTTCA-3′); *ii*) an *allele-specific reverse primer* for *R*. sp. I (270 bp amplicon) (*R*.sp. I_Rev, 5′-CTTGATTCAAATTGACATC-3′); *iii*) an *allele-specific reverse primer* for *R. sanguineus* s.s. (160 bp amplicon) (*R. s._*s.s._Rev, 5′-ACTGCACCTTGACTTAATATAA-3′) (Fig. 1).

To test the specificity of the assay, we performed multiplex PCRs using as a template the genomic DNA of known *R*. sp. I and *R. sanguineus* s.s. individuals^20^ and a mixture of their DNA. PCR was carried out in a 25 µl volume containing 5 ng of DNA, 10 mM Tris-HCl, pH 8.3, 2.0 mM MgCl_2_, 0.4 mM dNTPs, 0.4 µM of the common forward primer, 0.4 µM of each allele-specific reverse primer for *R*. sp. I, and *R. sanguineus* s.s., and 2.5 units of NZYTaq polymerase (NZYtech, Lisbon, Portugal). Negative controls containing all reagents but DNA were included in each PCR reaction to check for contaminations. PCR cycling conditions were as follow: 95 °C for 5 min followed by 34 cycles at 93 °C for 1 min, 55 °C for 1 min, 72 °C for 1 min 30 s, and a single final step at 72 °C for 10 min. The PCR products were then separated by electrophoresis run on 2% agarose and visualized by staining with Gelred (Sigma-Aldrich, Milan, Italy). The sizes of the DNA fragments were assessed using a 100 bp DNA ladder (Promega, Milan, Italy) run on the same gel. The reproducibility of the MAS-PCR assay was tested by performing three technical replicates of each reaction. To further support the specificity of our approach, the PCR products, after gel purification by NucleoSpin gel and PCR Clean-up purification kit (Macherey-Nagel, Düren, Germany), were double strand sequenced (https://www.gatc-biotech.com).

To assess the sensitivity of the MAS-PCR assay, the DNA of *R*. sp. I and *R. sanguineus* s.s. was mixed at different ratios. A total of 5 ng of DNA was used in each PCR reaction and the proportion of *R*. sp. I (or *R. sanguineus s.s*.) was serially diluted to 0.5, 0.1, 0.05 and 0.005 ng to obtain ratios of *R*. sp. I *vs R. sanguineus* s.s. DNA of 1:10, 1:50, 1:100, 1:1000.

### Screening of tick individuals

In a previous study, Dantas-Torres *et al*.^20^ realized pure and crossed lines using *R*. sp. I individuals from Italy (Putignano, Bari) and *R. sanguineus* s.s. individuals from Portugal (Faro)^20,46^. Briefly, ten unfed female and male ticks of the same lineage and of both lineages were placed on naïve rabbits to originate four experimental crosses: *i*) ♀ *R*. sp. I × ♂ *R*. sp. I; *ii*) ♀ *R. sanguineus* s.s. × ♂ *R. sanguineus* s.s.; *iii*) ♀ *R*. sp. I × ♂ *R. sanguineus* s.s. and *iv*) ♀ *R. sanguineus* s.s. × ♂ *R*. sp. I^20^.

In this paper, we developed a specific MAS-PCR assay and screened a total of 240 individuals from the above crossbreeding experiments, that included: all parental individuals (40 males and 40 females) originating the crosses; forty F1 offspring from each cross, including larvae, nymphs, adult females and males.

Genomic DNA was extracted from single ticks following Dantas-Torres *et al*.^20^, quantified using Nanodrop and diluted with sterile water to have a final concentration of 5 ng/µl. Genomic DNA (1 µl) from each individual was used as template for MAS-PCR following the amplification protocol and conditions described above.

Individuals from natural populations of *R*. sp. I and *R. sanguineus* s.s. were also analysed (Table 2). A total of 40 wild-caught individuals (10 larvae, 10 nymphs, 10 adult males and 10 unfed/non-mated adult females from the field) were analysed from the same sites where the individuals used for the cross-breeding experiments were collected^20^. *R*. sp. I ticks were collected from dogs living in a private shelter in Putignano (Bari, southern Italy), whereas *R. sanguineus s.s*. ticks were collected from privately owned dogs living in Faro (southern Portugal). All ticks were morphologically identified following Dantas-Torres *et al*.^21^. Genomic DNA was extracted from each individual and MAS-PCR assay was performed as described above. To check for consistency with MAS-PCR assay results a subset of parental and F1 ticks as well as wild-caught individuals were sequenced.

## Supplementary information

Original photos of the gels in Figures 2 and 3

## Data Availability

All data generated or analysed during this study are included in this published article.

## References

[1] Avise, J. C. *Molecular markers, natural history, and evolution* (2nd ed. Sunderland) (Sinauer Associates, 2004).

[2] Hickerson MJ (2010). Phylogeography’s past, present, and future: 10 years after. Mol Phyl Evol.

[3] Hebert PDN, Cywinska A, Ball SL, De Waard JR (2003). Biological identifications through DNA barcodes. Proc R Soc B.

[4] Berger TD, Shao R, Beati L, Miller H, Barker SC (2012). Phylogenetic analysis of ticks (Acari: Ixodida) using mitochondrial genomes and nuclear rRNA genes indicates that the genus *Amblyomma* is polyphyletic. Mol Phylogenet Evol.

[5] Berger TD, Shao R, Barker SC (2013). Phylogenetic analysis of the mitochondrial genomes and nuclear rRNA genes of ticks reveals a deep phylogenetic structure within the genus *Haemaphysalis*, and further elucidates the polyphyly of the genus *Amblyomma* with respect to *Amblyomma sphenodonti* and *Amblyomma elaphense*. Ticks Tick Borne Dis.

[6] Bendich AJ (2013). DNA abandonment and the mechanisms of uniparental inheritance of mitochondria and chloroplasts. Chromosome Res.

[7] Sato M, Sato K (2013). Maternal inheritance of mitochondrial DNA by diverse mechanisms to eliminate paternal mitochondrial DNA. Biochim Biophys Acta.

[8] Song WH, Ballard JW, Yi YJ, Sutovsky P (2014). Regulation of mitochondrial genome inheritance by autophagy and ubiquitin-proteasome system: implications for health, fitness, and fertility. Biomed Res Int.

[9] Greiner S, Sobanski J, Bock R (2015). Why are most organelle genomes transmitted maternally?. Bioessays.

[10] Ladoukakis D, Zouros E (2017). Evolution and inheritance of animal mitochondrial DNA: rules and exceptions. J Biol Res Thessaloniki.

[11] Breton S, Stewart DT (2015). Atypical mitochondrial inheritance patterns in eukaryotes. Genome.

[12] Polz MF, Cavanaugh CM (1998). Bias in template-to-product ratios in multitemplate PCR. Appl Environ Microbiol.

[13] Fontaine KM, Cooley JR, Simon C (2007). Evidence for Paternal Leakage in Hybrid Periodical Cicadas (Hemiptera: Magicicada spp.). Plos One.

[14] White DJ, Wolff JN, Pierson M, Gemmell NJ (2008). Revealing the hidden complexities of mtDNA inheritance. Mol Ecol.

[15] Dantas-Torres F, Chomel BB, Otranto D (2012). Ticks and tick-borne diseases: a One Health perspective. Trends Parasitol.

[16] Epis S (2014). Temporal dynamics of the ABC transporter response to insecticide treatment: insights from the malaria vector *Anopheles stephensi*. Sci Rep.

[17] Rizzoli A (2014). *Ixodes ricinus* and its transmitted pathogens in urban and peri-urban areas in Europe: new hazards and relevance for public health. Front Public Health.

[18] Cafarchia C (2015). Potential role of ATP-binding cassette transporters against acaricides in the brown dog tick *Rhipicephalus sanguineus* sensu lato. Med Vet Entomol..

[19] Noriega JA (2018). Research trends in ecosystem services provided by insects. Basic Appl Ecol.

[20] Dantas-Torres F (2018). Biological compatibility between two temperate lineages of brown dog ticks, *Rhipicephalus sanguineus* (*sensu lato*). Parasit Vector.

[21] Dantas-Torres F (2013). Morphological and genetic diversity of *Rhipicephalus sanguineus* sensu lato from the New and Old Worlds. Parasit Vector.

[22] Wolff JN, Nafisinia M, Sutovsky P, Ballard JWO (2013). Paternal transmission of mitochondrial DNA as an integral part of mitochondrial inheritance in metapopulations of *Drosophila simulans*. Heredity.

[23] Gemmell NJ, Metcalf VJ, Allendorf FW (2004). Mother’s curse: the effect of mtDNA on individual fitness and population viability. Trends Ecol Evol.

[24] Hill GE (2015). Mitonuclear ecology. Mol Biol Evol.

[25] Barton NH, Hewitt GM (1985). Analysis of hybrid zones. Annu Rev Ecol Syst.

[26] Porretta D, Urbanelli S (2012). Evolution of premating reproductive isolation among conspecific populations of the sea rock-pool beetle *Ochthebius urbanelliae* driven by reinforcing natural selection. Evolution.

[27] Abbott R (2013). Hybridization and speciation. J Evol Biol.

[28] Urbanelli S (2014). Hybridization, natural selection and evolution of reproductive isolation: a 25-years survey of an artificial sympatric area between two mosquito sibling species of the *Aedes mariae* complex. Evolution.

[29] Bisconti R, Porretta D, Arduino P, Nascetti G, Canestrelli D (2018). Hybridization and extensive mitochondrial introgression among fire salamanders in peninsular Italy. Sci Rep.

[30] Mastrantonio V, Porretta D, Urbanelli S, Crasta G, Nascetti G (2016). Dynamics of mtDNA introgression during species range expansion: insights from an experimental longitudinal study. Sci Rep.

[31] Garcia-Elfring A (2017). Admixture on the northern front: Population genomics of range expansion in the white-footed mouse (*Peromyscus leucopus*) and secondary contact with the deer mouse (*Peromyscus maniculatus*). Heredity.

[32] Latrofa MS, Dantas-Torres F, Giannelli A, Otranto D (2014). Molecular detection of tick-borne pathogens in *Rhipicephalus sanguineus* group ticks. Ticks Tick Borne Dis.

[33] Porretta D (2016). Exon-intron structure and sequence variation of the calreticulin gene among *Rhipicephalus sanguineus* group ticks. Parasit Vector.

[34] Hornok S (2017). East and west separation of *Rhipicephalus sanguineus* mitochondrial lineages in the Mediterranean Basin. Parasit Vector.

[35] Rokas A, Ladoukakis E, Zouros E (2003). Animal mitochondrial DNA recombination revisited. Trends Ecol Evol.

[36] Boyce TM, Zwick ME, Aquadro CF (1989). Mitochondrial DNA in the bark weevils: Size, structure and heteroplasmy. Genetics.

[37] Dantas-Torres F (2018). Species Concepts: What about Ticks?. Trends Parasitol.

[38] Volz-Lingenhohl A, Solignac M, Sperlich D (1992). Stable heteroplasmy for a large-scale deletion in the coding region of *Drosophila subobscura* mitochondrial DNA. Proc Natl Acad Sci USA.

[39] Kann LM, Rosenblum EB, Rand DM (1997). Aging, mating, and the evolution of mtDNAheteroplasmy in *Drosophila melanogaster*. Proc Natl Acad Sci USA.

[40] Nardi F, Caraoelli A, Fanciulli PP, Dallai R, Frati F (2001). The Complete mitochondrial DNA sequence of the basal hexapod *Tetrodontophora bielanensis*: Evidence for heteroplasmy and tRNA translocations. Mol Biol Evol.

[41] Marcadé I (2007). Structure and evolution of the atypical mitochondrial genome of *Armadillidium vulgare* (Isopoda, Crustacea). J Mol Evol.

[42] Leeuwen TV (2008). Mitochondrial heteroplasmy and the evolution of insecticide resistance: Non-Mendelian inheritance in action. Proc Natl Acad Sci USA.

[43] Xiong H, Barker SC, Burger TD, Raoult D, Shao R (2013). Heteroplasmy in the mitochondrial genomes of human lice and ticks revealed by high throughput sequencing. PLoS One.

[44] Nunes MDS, Dolezal M, Schlötterer C (2013). Extensive paternal mtDNA leakage in natural populations of *Drosophila melanogaster*. Mol Ecol.

[45] Robison GA, Balvin O, Schal C, Vargo EL, Booth W (2015). Extensive mitochondrial heteroplasmy in natural populations of a resurging human pest, the bed bug (Hemiptera: Cimicidae). J Med Entomol.

[46] Dantas-Torres F (2017). Genetic characterization of *Rhipicephalus sanguineus* (*sensu lato*) ticks from dogs in Portugal. Parasit Vector.

[47] Dokianakis E, Ladoukakis ED (2014). Different degree of paternal mtDNA leakage between male and female progeny in interspecific *Drosophila* crosses. Ecol Evol.

[48] Arunkumar KP, Metta M, Nagaraju J (2006). Molecular phylogeny of silkmoths reveals the origin of domesticated silkmoth, *Bombyx mandarin*a and paternal inheritance of *Antheraea proley*i mitochondrialDNA. Mol Phyl Evol.

[49] Sherengul W, Kondo R, Matsuura ET (2006). Analysis of paternal transmission of mitochondrial DNA in *Drosophila*. *Genes*. Gen Syst.

[50] Kondo R (1990). Incomplete Maternal Transmission of Mitochondrial DNA in *Drosophila*. Genetics.

[51] Lansman RA, Avise JC, Huettel MD (1983). Critical Experimental Test of the Possibility of “Paternal Leakage” of Mitochondrial DNA. Proc Natl Acad Sci USA.

[52] Gawande SJ (2017). Heteroplasmy due to coexistence of mtCOI haplotypes from different lineages of the *Thrips tabaci* cryptic species group. Bull Entomol Res.

[53] Kastally C, Mardulyn P (2017). Widespread co-occurrence of two distantly related mitochondrial genomes in individuals of the leaf beetle *Gonioctena intermedia*. Biol Letters.

[54] Gantenbein B, Fet V, Gantenbein-Ritter I, Balloux F (2005). Evidence for recombination in scorpioon mitochondrialDNA (Scorpiones: Buthidae). Proc Royal Soc B.

[55] Andolfatto P, Scriber JM, Charlesworth B (2003). No association between mitochondrial DNA haplotypes and a female-limited mimicry phenotype in *Papilio glaucus*. Evolution.

[56] Meusel MS, Moritz RFA (1993). Transfer of paternal mitochondrial DNA during fertilization of honeybee (*Apis mellifera* L.) eggs. *Curr*. Gen.

