# Supplementary material for: Paternal leakage and mtDNA heteroplasmy in Rhipicephalus spp. ticks
Source: Sci Rep. 2019 Feb 6;9:1460. doi: 10.1038/s41598-018-38001-8 (PMC6365633; doi:10.1038/s41598-018-38001-8)

Paternal leakage and mtDNA heteroplasmy in *Rhipicephalus* spp. ticks

Valentina Mastrantonio[1†], Maria Stefania Latrofa[2†], Daniele Porretta[1]*, Riccardo Paolo Lia[2], Antonio Parisi[3], Roberta Iatta[2], Filipe Dantas-Torres[2,4], Domenico Otranto[2], Sandra Urbanelli[1]

[1] Department of Environmental Biology, Sapienza University of Rome, Rome, Italy

[2] Department of Veterinary Medicine, University of Bari, 70010 Valenzano, Bari, Italy

[3] Istituto Zooprofilattico Sperimentale della Puglia e della Basilicata, Contrada S. Pietro Piturno, 70017 Putignano, Bari, Italy

[4] Department of Immunology, Aggeu Magalhães Institute, Oswaldo Cruz Foundation, 50670420 Recife, Pernambuco, Brazil

*Corresponding author: daniele.porretta@uniroma1.it

[†] These authors equally contributed to the paper

Supplementary figure 1. Original photos of the gels that are shown in the Figure 2.

Figure 2A

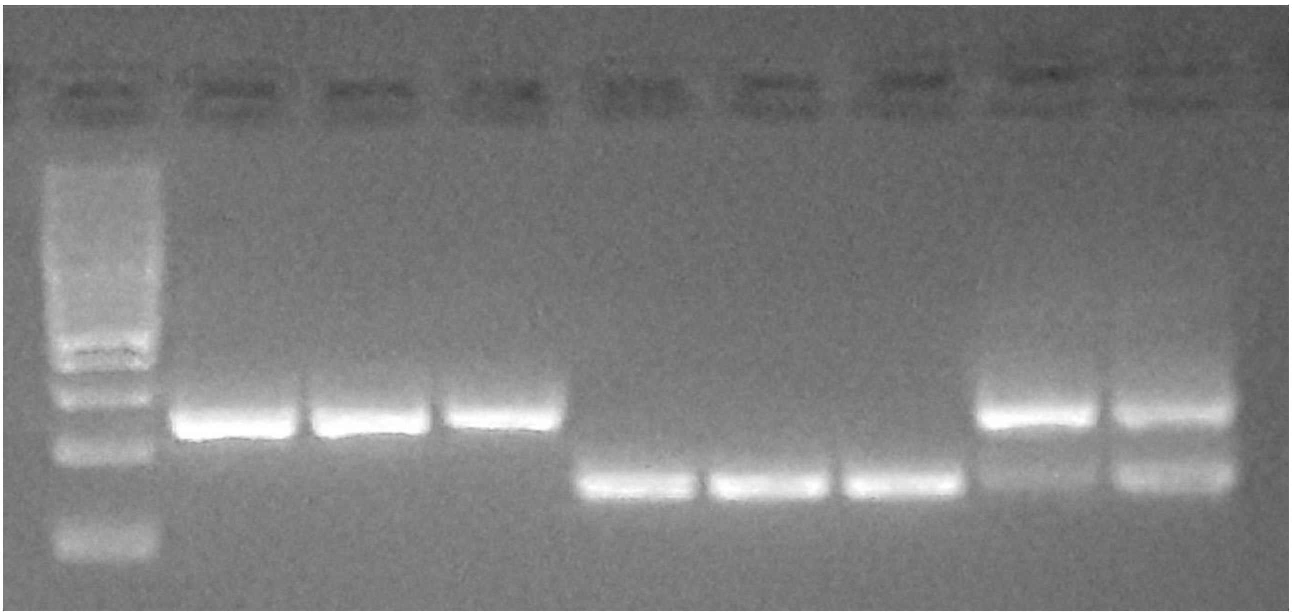

Figure 2B

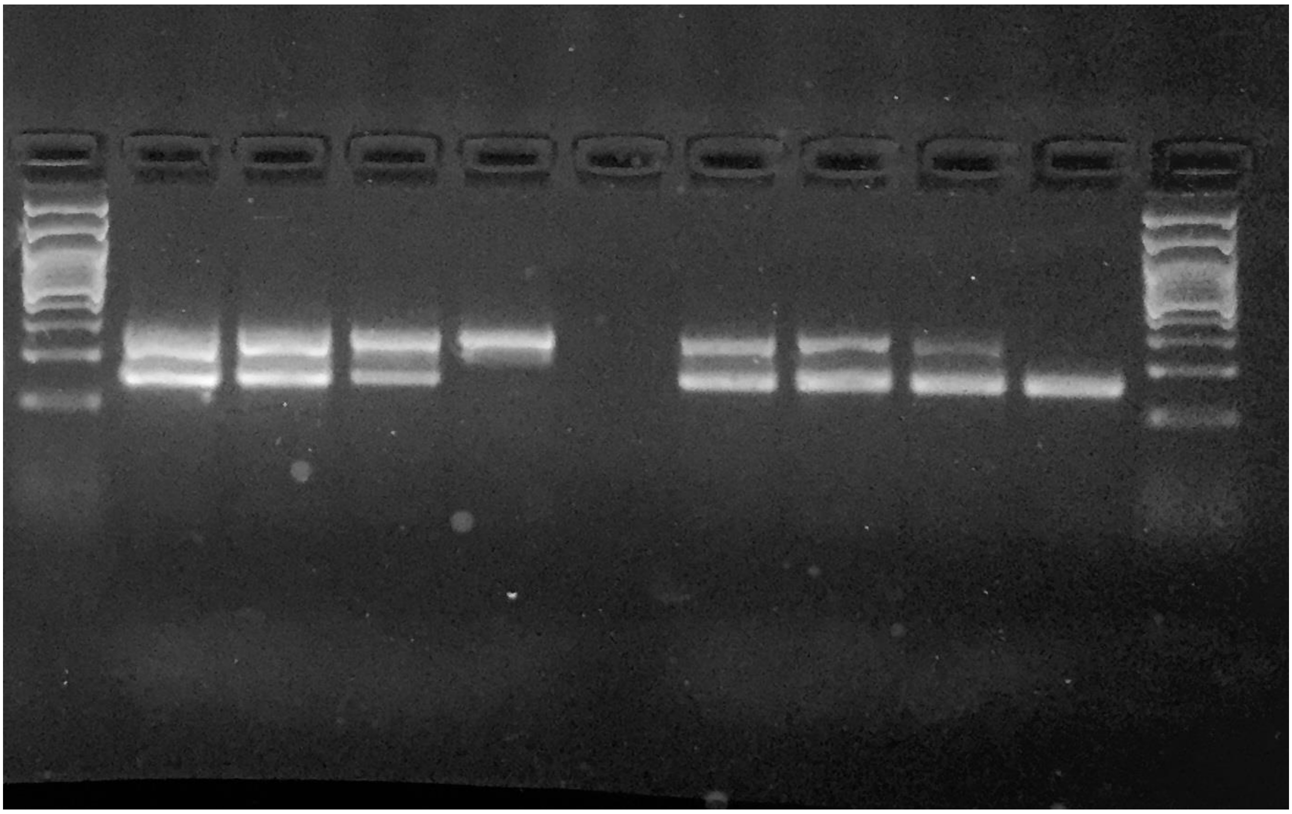

Supplementary figure 2. Original photo of the gels that is shown in the Figure 3.

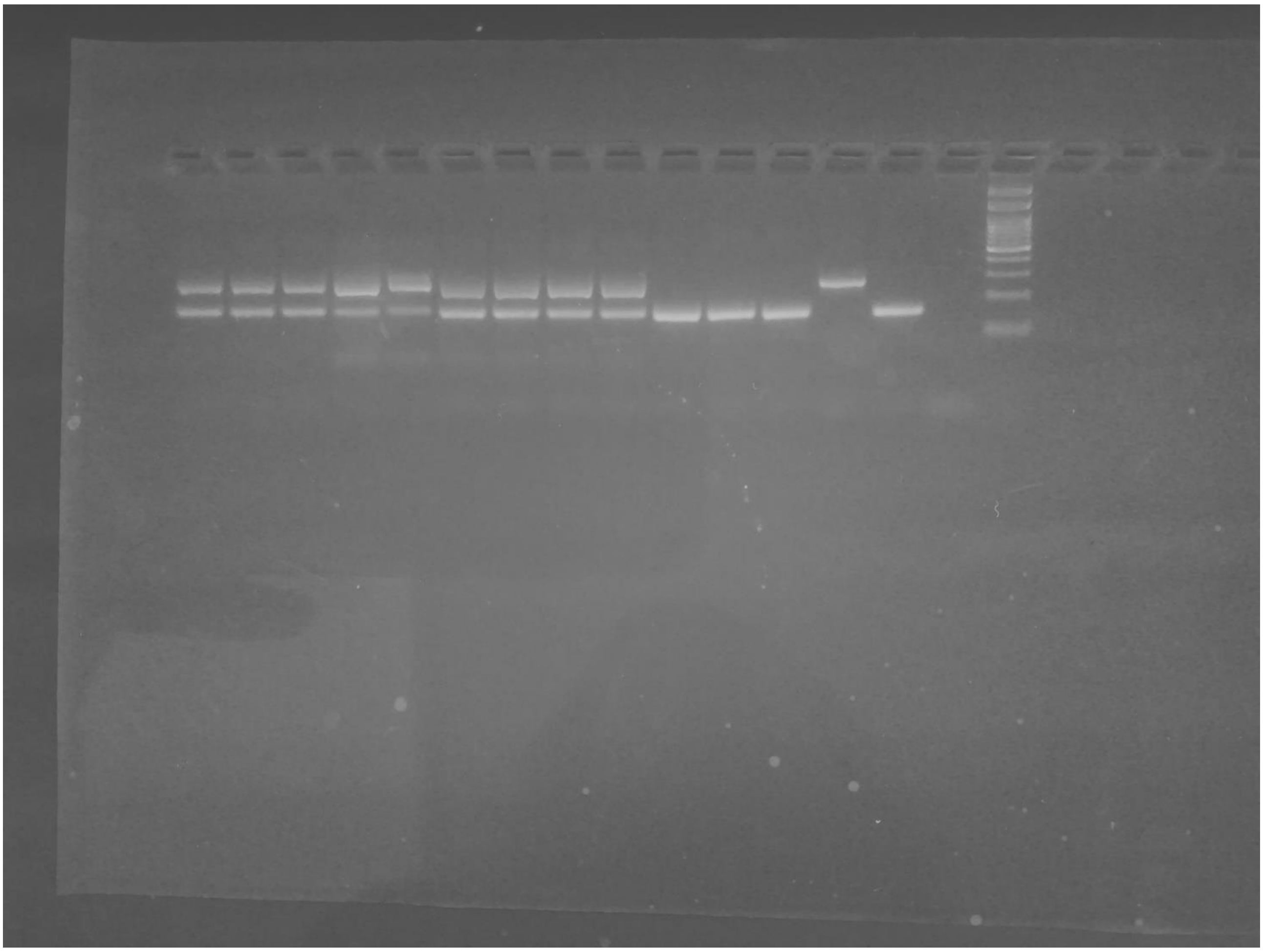

Supplement: Supplementary file 1 — Original photos of the gels in Figures 2 and 3 [file 41598_2018_38001_MOESM1_ESM.pdf]
